# A Prospective Cohort Study Evaluating the Ability of Anticipated Pain, Perceived Analgesic Needs, and Psychological Traits to Predict Pain and Analgesic Usage following Cesarean Delivery

**DOI:** 10.1155/2016/7948412

**Published:** 2016-04-07

**Authors:** Brendan Carvalho, Ming Zheng, Scott Harter, Pervez Sultan

**Affiliations:** ^1^Department of Anesthesia, Stanford University School of Medicine, Stanford, CA 94303, USA; ^2^Department of Obstetric Anesthesiology, Ministry Saint Michael's Hospital, 900 Illinois Avenue, Stevens Point, WI 54481, USA; ^3^Department of Anaesthesia, University College Hospital, London NW1 2BU, UK

## Abstract

*Introduction*. This study aimed to determine if preoperative psychological tests combined with simple pain prediction ratings could predict pain intensity and analgesic usage following cesarean delivery (CD).* Methods*. 50 healthy women undergoing scheduled CD with spinal anesthesia comprised the prospective study cohort. Preoperative predictors included 4 validated psychological questionnaires (Anxiety Sensitivity Index (ASI), Fear of Pain (FPQ), Pain Catastrophizing Scale, and Eysenck Personality Questionnaire) and 3 simple ratings: expected postoperative pain (0–10), anticipated analgesic threshold (0–10), and perceived analgesic needs (0–10). Postoperative outcome measures included post-CD pain (combined rest and movement) and opioid used for the 48-hour study period.* Results*. Bivariate correlations were significant with expected pain and opioid usage (*r* = 0.349), anticipated analgesic threshold and post-CD pain (*r* = −0.349), and perceived analgesic needs and post-CD pain (*r* = 0.313). Multiple linear regression analysis found that expected postoperative pain and anticipated analgesic needs contributed to post-CD pain prediction modeling (*R* ^2^ = 0.443, *p* < 0.0001); expected postoperative pain, ASI, and FPQ were associated with opioid usage (*R* ^2^ = 0.421, *p* < 0.0001).* Conclusion*. Preoperative psychological tests combined with simple pain prediction ratings accounted for 44% and 42% of pain and analgesic use variance, respectively. Preoperatively determined expected postoperative pain and perceived analgesic needs appear to be useful predictors for post-CD pain and analgesic requirements.

## 1. Introduction

Cesarean delivery (CD) is associated with moderate to severe pain that is often incompletely relieved by modern pain management protocols [1]. A large variability in postoperative pain and analgesic use exists among patients undergoing surgery, and women with severe acute post-CD pain have an increased risk of persistent incisional pain compared to patients with mild acute postoperative pain [2]. The ability to preoperatively identify patients “at risk” of developing severe postoperative pain and higher analgesic dose requirement would be beneficial, potentially facilitating the use of individualized or stratified analgesic treatment plans. Patients deemed to be at high risk of developing severe postoperative pain, for example, could prophylactically receive larger intrathecal neuraxial opioid doses and/or additional adjunctive medications such as gabapentin or clonidine, which are otherwise not routinely utilized for CD due to their side-effect profiles [3, 4].

Psychological predictors of surgical pain and analgesic requirements are incompletely understood. A number of psychological characteristics (anxiety, pain catastrophizing, and fear of pain) have been shown to significantly correlate with postoperative pain [3, 5, 6]. Evaluating these psychological characteristics however requires time-consuming questionnaires and additional trained personnel making them impractical for routine clinical use. A robust, quick to perform, point-of-care set of questions that accurately predict postoperative pain may improve pain management after CD. Simple ratings of patients' anticipated pain and analgesic needs have been found to correlate moderately with post-CD evoked pain intensity [7], but these simplified ratings were not evaluated alongside comprehensive validated psychological tests.

The aim of this study was to determine whether preoperative psychological characteristics (anxiety, pain catastrophizing, fear of pain, and personality traits) measured using validated questionnaires and simplified anticipated pain and analgesic expectation rating scores reliably predict pain intensity and analgesic use following elective CD. We hypothesized that these psychological tests combined with simplified anticipated pain and analgesic expectation rating scores would predict women's pain and analgesic use following surgery.

## 2. Materials and Methods

### 2.1. Study Design and Patient Population

Healthy pregnant women aged 18–45 years with a singleton term (>37 weeks of gestation) pregnancy scheduled for elective CD without postpartum tubal ligation were enrolled in this Institutional Review Board-approved, prospective, cohort study. Consecutive patients who fulfilled study criteria were approached to participate in the study and signed written consents on the day of surgery at their preoperative anesthetic evaluation within 2 hours prior to their CD. The study was conducted at Lucile Packard Children's Hospital Stanford, California. Exclusion criteria for this prospective cohort study included significant medical or obstetric disease; multiple gestation; inability to understand English; contraindication to neuraxial anesthesia; failed neuraxial anesthesia requiring conversion to general anesthesia; chronic opioid or antidepressant use; recent (less than 48 hours) analgesic medication use; intolerance or allergy to opioids, nonsteroidal anti-inflammatories, or local anesthetics.

### 2.2. Predictive Psychological and Expectation Questionnaires

Four validated psychological questionnaires were given to women after the initial interview: (1) Anxiety Sensitivity Index (ASI) which assesses anxiety associated with potentially unpleasant events [8]; (2) Fear of Pain Score (FPQ III) which evaluates fear that is generated from physical insult or injury [9, 10]; (3) Pain Catastrophizing Scale (PCS) with three components (rumination, magnification, and helplessness) scored separately and totaled [11]; (4) Eysenck Personality Questionnaire Revised-Short Scale (EPQR-S) with questions related to psychoticism, extroversion, neuroticism, and lying [12, 13].

A questionnaire to determine women's expected postoperative pain, anticipated analgesic threshold, and perceived analgesic needs was distributed and completed by study participants at the same time as the psychological questionnaires. Specifically, we asked the following: (1) expected postoperative pain: “how much pain do you expect to experience after your surgery on a pain scale of 0–10? (0 = no pain, 10 = worse pain imaginable)”; (2) anticipated analgesic threshold: “at what point on a pain scale of 0–10 would you likely request postoperative pain relief? (0 = no pain, 10 = worse pain imaginable)”; and (3) perceived analgesic needs: “what do you expect your analgesic requirements will be after surgery? (0 = no analgesia, 10 = highest amount).” Women having previously undergone surgery and/or CD were asked to rate their most physically painful life experience and current pain using a numerical verbal pain score (NVPS 0–10: 0 = no pain, 10 = worse pain imaginable). Patient demographic and obstetric data were also obtained during the initial interview.

### 2.3. Study Protocol

All patients received spinal anesthesia with intrathecal hyperbaric bupivacaine 12 mg, fentanyl 10 mcg, and morphine 100 mcg. Postoperative pain was managed with oral ibuprofen 600 mg every 6 hours for 48 hours. Oral oxycodone/hydrocodone 5 mg with acetaminophen 325 mg for pain ≤4/10 and oral oxycodone/hydrocodone 10 mg with acetaminophen 650 mg for pain >4/10 were available if requested for breakthrough pain. Up to 10 mg of oxycodone/hydrocodone every 4 hours with a maximum dose of 60 mg oxycodone/hydrocodone or 4 g acetaminophen in a 24-hour period was allowed. Intravenous morphine 4 mg (maximum 10 mg every hour) was reserved for severe pain or pain resistant to oral opioids. Postoperative analgesics were offered in the postanesthesia care unit and postpartum ward.

### 2.4. Outcome Response Measures

The primary outcome response measures were postoperative pain and analgesic consumption. Post-CD pain at rest and on movement (defined as sitting upright at 90 degrees) was measured using a NVPS 0–10 at 6, 24, and 48 hours and analyzed as NVPS over time area-under-the-curve (AUC) over the 48-hour study period. The total amount of supplemental opioid analgesic medication (oral and intravenous) used in the 48-hour study period was determined in morphine mg equivalents. Oxycodone/hydrocodone were converted to morphine mg equivalents for analysis using a standardized opioid conversion, with oral oxycodone/hydrocodone 20 mg being considered equivalent to 10 mg intravenous morphine [14]. Time to first analgesic request was defined as minutes from end of surgery and length of hospital stay as hours from end of surgery and to hospital discharge. Satisfaction with analgesia (0 = totally unsatisfied and 100 = totally satisfied) was determined at 48 hours after CD, and the length of hospital stay (in hours) was recorded. Decisions regarding patient discharge were made by obstetricians not involved in the study. NVPS at the incision site at 1-week after CD was also determined. Investigators collecting outcome response data were blinded from the preoperative predictor test and questionnaire results.

### 2.5. Statistical Analysis

Demographic and outcome data are summarized with descriptive statistics and expressed as the mean ± standard deviation, median [interquartile range], and number (percentage) as appropriate. To check which pairs showed significant correlations, bivariate analysis (measured by Spearman's rho statistic) was initially performed for each of the predictors and outcome response pairs, and the method described by Benjamini and Hochberg [15] was used to adjust for false-discovery-rate when multiple hypothesis testings were performed simultaneously. Adjusted *p* value < 0.05 was considered statistically significant.

To achieve as close linearity as possible between a predictor and outcome response measurement transformation was performed to predictors, and the transformation that led to the largest absolute correlation between the transformed predictor and the response was used for multivariate linear regression analysis. A forward-backward model selection method was selected for the multivariate linear regression modeling. To minimize the problem of overfitting, a selection method based on Akaike Information Criterion (AIC) [16] was used for each response variable to determine the best model. This model selection starts with a null model with no variables in the model, and, after each iteration, the model considers whether adding any variable that was not in the model or eliminating any variable that was currently included in the model would lead to smaller AIC score [17]. Statistical significance was assessed for this final model only and considered significant at a *p* value ≤ 0.01. Using this model selection method, multicollinearity among predictors was minimal. The statistical assumption of the multivariate linear regression model was graphically examined, and Shapiro-Wilk's test for normality was applied to ensure that residuals were normally distributed and had roughly constant mean and variance across the entire range of the fitted values. Multivariate linear regression modeling was repeated using only the three simple ratings (expected postoperative pain, anticipated analgesic threshold, and perceived analgesic needs) to explore the predictive value of these combined variables.

Subjects with missing values in at least one predictor were excluded for multiple regression analysis. To avoid spurious findings due to outliers, outlying values for the response variable were excluded during the regression analysis. Outlying values were determined by the following definition: below Q1 − 1.5 *∗* IQR or above Q3 + 1.5 *∗* IQR (where Q1 and Q3 are the 1st and 3rd quartile, resp., and IQR is the “Interval Quartile Range” and defined as Q3-Q1). The number of outliers removed for postoperative opioid use and the combined rest and movement NVPS AUC were 2 and 0, respectively. The analysis was performed using the statistics software R (https://www.r-project.org/) and IBM SPSS Version 20 (Armonk, New York).

## 3. Results

Fifty patients were enrolled and completed the study, with no patients lost to follow-up or withdrawn during the 48-hour study period. Fourteen subjects had missing values in at least one predictor and were therefore excluded for multiple regression analysis. Demographic and obstetric data of the study cohort are presented in Table 1.

Bivariate correlations among the preoperative psychological questionnaires and outcome response measures of opioid used and pain scores are shown in Table 2. There were no significant correlations with ASI, FPQ, PCS, and any Eysenck Personality traits (psychoticism, extroversion, neuroticism, and lying) with any outcome measures. Postoperative opioid usage correlated with expected postoperative pain (*r* = 0.349, Table 2). Combined (Rest + Move) NVPS AUC correlated with anticipated analgesic threshold (*r* = −0.349, Table 2) and perceived analgesic needs (*r* = 0.313, Table 2).

Multiple linear regression analysis of the all preoperative predictors and key clinical outcome measures following CD are displayed in Table 3(a). The outcome of postoperative opioid usage was predicted with ASI, FPQ, and expected postoperative pain contributing to the model (*R* ^2^ = 0.421, *p* = 0.0002; Table 3(a)). *R* ^2^ decreased to 0.21 (*p* = 0.0027) when only the three simple ratings preoperative predictive tests (expected postoperative pain, anticipated analgesic threshold, and perceived analgesic needs) were utilized in the multiple linear regression modeling (Table 3(b)). The outcome of combined (Rest + Move) NVPS AUC was predicted with the expected postoperative pain and perceived analgesic needs contributed to the modeling; *R* ^2^ = 0.443, *p* = 0.00002 with all predictive variables considered (Table 3(a)) and *R* ^2^ = 0.447, *p* = 0.00001 when only the three simple ratings preoperative predictive tests (Table 3(b)) were utilized in the multiple linear regression modeling.

Correlations among the various psychological questionnaires and ratings are outlined in Table 4. There were several strong correlations between ASI, FPQ, and PCS (Table 4). Psychoticism correlated strongly with FPQ (*r* = −0.56, *p* < 0.05) and PCS (*r* = −0.45, *p* < 0.05). There were no significant correlations among the Eysenck Personality traits (psychoticism, extroversion, neuroticism, and lying). The mean ± SD pain scores after CD at rest were 2 ± 2, 2 ± 2, and 2 ± 1 at 6, 24, and 48-hour time points. The mean ± SD post-CD pain scores on sitting were 4 ± 2, 4 ± 1, and 3 ± 2 at 6, 24, and 48-hour time points. The mean ± SD analgesic consumption (mg morphine equivalents) over the 48-hour study period was 29 ± 19. The time to first analgesic request was 110 [93–136] minutes. The median [IQR] patient satisfaction with analgesia was 90 [90–100] at 48 hours postcesarean delivery. The median [IQR] length of hospital stay was 98 [76–99] hours and the median [IQR] incisional reported pain at 1 week was 2 [1–3].

## 4. Discussion

Findings from this study show that preoperative psychological tests combined with simple pain prediction ratings accounted for 44% and 42% of postoperative pain and analgesic usage variance, respectively. The simple scaled ratings of patients' expected postoperative pain (how much pain do you expect to experience after your surgery on a pain scale of 0–10?), anticipated analgesic threshold (at what point on a pain scale of 0–10 would you likely request postoperative pain relief?), and perceived analgesic needs (what do you expect your analgesic requirements will be after surgery?) were useful in predicting the post-CD pain experience. The finding that a simple, quick to perform, point-of-care set of questions may help predict postoperative pain after CD is encouraging, especially considering that questionnaires evaluating anxiety, pain catastrophizing, fear of pain, and personality traits are time-consuming and therefore not well suited to the clinical demands of the perioperative setting.

The three simple ratings of patients' expected postoperative pain, anticipated analgesic threshold, and perceived analgesic needs determined prior to CD were able to predict 45% of the variability of postcesarean pain and 21% of the opioid used. Pan et al.'s [7] study found that scores from three simple preoperative screening questions on surgical anxiety (0–100), anticipated pain (0–100), and anticipated pain medication need (0–5) accounted for 20% of the variance in postcesarean evoked pain. They found that these three screening questions demonstrated a sensitivity and specificity of 0.69 and 0.69, respectively, for identifying parturients in the top 20th percentile for activity-associated post-CD pain [7]. We have previously found that simple anxiety rating scores (0–100) were not useful in predicting the labor pain experience; nor did they correlate with a detailed anxiety assessment tool, the ASI [17]. In a labor setting, self-reported ratings of expected analgesic requirement (0 = no analgesia, 10 = highest possible amount) correlated with both time to epidural request (*r* = −0.34, *p* < 0.05) and labor pain (*r* = −0.37, *p* < 0.05) [17]. The optimal simple screening tool to determine postoperative or labor pain and analgesic needs is yet to be determined. This study and the results from Pan et al.'s study [7] are encouraging and suggest that these simple patient-perceived anticipated pain rating scores may be useful in predicting the post-CD pain experience.

Under conditions of the study, psychological characteristics (anxiety, pain catastrophizing, fear of pain, and personality traits) in isolation were of limited predictive value for post-CD pain. In previous nonobstetric studies, many of these psychological tests, in particular pain catastrophizing, fear of pain, and anxiety, have correlated with postoperative pain and analgesic usage [3, 5, 18, 19]. In a CD setting, studies have found that pain catastrophizing and anxiety correlate with postoperative pain [20–22]. Strulov et al. [20] demonstrated that pain, not analgesic consumption, on the second postoperative day was predicted by preoperative pain catastrophizing (*R* ^2^ = 0.139, *p* = 0.021). Granot and Ferber [21] found that Pain Catastrophizing Scales and State-Trait Anxiety Inventory scores significantly correlated with postoperative pain scores and opioid usage. State-Trait Anxiety Inventory has also previously been shown to correlate with recovery room analgesia (*R* ^2^ = 0.27, *p* < 0.01) and total analgesic needs (*R* ^2^ = 0.22, *p* < 0.01) following CD [22].

The impact of personality on postoperative pain is an underexplored area. Different personality traits may impact different measures of pain and analgesic needs and may differ depending on the clinical setting. Neuroticism has previously been found to be a marker of vulnerability to functional gastrointestinal disorder [23], as well as arthritis-related pain self-efficacy beliefs and pain control appraisals [24]. In the labor setting, we previously found no significant bivariate correlations between extroversion, neuroticism, lying, and psychoticism and any labor pain and epidural local anesthetic use outcomes [17]; however, psychoticism and extroversion did contribute to the predictive regression modeling of pain at epidural request and labor pain burden [17]. In the current study, none of the Eysenck Personality traits (extroversion, neuroticism, lying, and psychoticism) correlated significantly with any post-CD analgesic outcome measures, and the ability of personality traits to predict postoperative pain appears to be limited.

The predictive value of many of these psychological characteristics has been limited to only a small percentage of postoperative pain variance [3, 5]. Psychological factors may however enhance the ability for experimental pain tests to predict surgical pain [3, 25–27]. In studies of the CD population, anxiety and pain catastrophizing measurements enhance the ability of experimental pain tests to predict postoperative pain and analgesic usage [20, 22, 28].

There are potentially a number of limitations to this study. Although we measured a number of psychological factors, we acknowledge that some psychological and social factors were not assessed. We selected anxiety, fear of pain, and pain catastrophizing because these factors have been previously found to be associated with pain in the surgical setting. Although the psychological questionnaires are validated, they are not specific to cesarean delivery; nor have they all been validated in surgical settings. We did not assess women's baseline mood and acknowledge that postoperative depression has been found to be associated with acute postsurgical pain intensity [29] and persistent postpartum pain [30]. We did not assess sleep disturbance and appreciate chronic sleep deprivation may be associated with postoperative pain [31]. We chose to assess pain and analgesic usage as our primary outcome measures to reflect the post-CD pain experience. We acknowledge that these outcome measures may not completely represent or reflect the entire cesarean delivery pain experience. The time to analgesic request and maternal satisfaction were not assessed. Additionally, we only measured acute pain and analgesic outcomes; both psychological characteristics and personality traits may impact chronic pain reporting and pain experienced. The sample population (elective cesarean delivery in healthy women) without exclusion criteria (e.g., no chronic pain) limits the generalizability of the study results. Our sample size of 50 with 14 having incomplete data limited our ability to study outcome measures beyond pain and analgesic use, required us to combine rest and activity pain into one outcome measure, restricted predictive measures that could be studied, and limited direct comparisons among various predictors. Pain scores of the study cohort were also low due to the use of multimodal analgesic management.

In conclusion, our study demonstrates that preoperative psychological tests combined with simple pain prediction ratings explained 44% and 42% of post-CD pain and analgesic usage variance, respectively. Psychological questionnaires (ASI, FPQ, PCS, and EPQR-S personality traits) in isolation showed weak bivariate correlations with pain and analgesic usage after CD; however, these questionnaires did contribute to the multivariate prediction modeling. Simple, quick to perform rating questions determining patients' expected postoperative pain, anticipated analgesic threshold, and perceived analgesic needs appear to be useful for postoperative pain and analgesic usage prediction, accounting for 45% and 21% of the observed variance in post-CD pain and opioid use, respectively. Findings from this study demonstrate the importance of asking patients' expected postoperative pain and perceived analgesic needs prior to CD to better anticipate postoperative pain and analgesic use, and this may potentially facilitate individualized analgesic treatment protocols based on patient's perceived needs. Future studies are required to determine the optimal simple screening tools to determine postoperative pain and analgesic needs and to study whether alterations made to analgesic protocols based on patients' preoperative expectations improve analgesic outcomes.

## Figures and Tables

**Table 1:** Demographic and obstetric data of the cesarean delivery study population (*n* = 50).

Age (years)	35 ± 4
Body Mass Index (kg/m^2^)	30 ± 4
Race	
Caucasian	27 (54)
Asian	15 (30)
Other	8 (16)
Nulliparous (%)	20%
Gestational age	39 [38–39]
Reason for cesarean	
Previous cesarean	35 (70)
Breech	5 (10)
Other	10 (20)

Values expressed as mean ± SD, mean [IQR], and number (percentage) as appropriate.

**Table 2:** Bivariate correlations between preoperative predictive tests and response outcome measures postcesarean delivery.

	Opioid used^a^	Combined (Rest + Move) NVPS AUC^b^
ASI	−0.019	0.148
FPQ	−0.132	0.165
PCS	−0.137	0.067
Extroversion	0.094	0.126
Neuroticism	0.23	0.127
Lying	−0.207	0.061
Psychoticism	0.114	−0.09
Expected postoperative pain^1^	0.349^†^	0.263
Anticipated analgesic threshold^2^	0.032	−0.349^†^
Perceived analgesic need^3^	0.169	0.313^†^

^†^Unadjusted *p* value < 0.05.

AUC = area-under-the curve; NVPS = numerical verbal pain score (0–10, 0 = no pain, 10 = worst pain imaginable).

ASI = Anxiety Sensitivity Index; FPQ = Fear of Pain Score III; PCS = Pain Catastrophizing Scale.

The personality categories (psychoticism, extroversion, neuroticism, and lying) were derived from Eysenck Personality Questionnaire Revised-Short Scale (EPQR-S).

^a^Total amount of supplemental opioid analgesics (oral and intravenous) used in 48 hours was determined by adding intravenous morphine doses to oxycodone/hydrocodone (converted to morphine mg equivalents for analysis with oral oxycodone/hydrocodone 20 mg being considered equivalent to 10 mg IV morphine [14]).

^b^NVPS at rest and at movement (defined as sitting upright at 90 degrees) was measured at 6, 24, and 48 hours, and the pain burden was determined as NVPS over time AUC over the 48-hour study period.

^1^Expected postoperative pain: “how much pain do you expect to experience after your surgery on a pain scale of 0–10?”

^2^Anticipate analgesic threshold: “at what point on a pain scale of 0–10 would you likely request post-op pain relief?”

^3^Perceived analgesic need: “what do you expect your analgesic requirements will be after surgery? (0 = no analgesia, 10 = highest possible amount).”

**(a):** 

Response outcome^a^	*R* ^2^ coefficient	*p* value	Predictor tests in model
Opioid used^b^	0.421	0.0002	9.36 – 9.97 *∗* ASI – 6.9 × 10^−6^ *∗* FPQ + 0.023 *∗* expected pain^1^
Combined (Rest + Move) NVPS AUC^c^	0.443	0.00002	57.68 + 108.96 *∗* expected pain^1^ – 1.36 *∗* perceived analgesic need^2^

AUC = area-under-the curve; NVPS = numerical verbal pain score (0–10, 0 = no pain, 10 = worst pain imaginable).

ASI = Anxiety Sensitivity Index; FPQ = Fear of Pain Score III; PCS = Pain Catastrophizing Scale.

The personality categories (psychoticism, extroversion, neuroticism, and lying) were derived from Eysenck Personality Questionnaire Revised-Short Scale (EPQR-S).

^a^Transformations used were as follows.

For opioid used: ASI (1 + *x*)^−0.3^, FPQ: *x* ^3^, and expected postoperative pain: *x* ^2.8^.

For combined (Rest + Move) NVPS AUC: expected postoperative pain: *x* ^0.5^, perceived analgesic need: *x* ^2.3^.

^b^Total amount of supplemental opioid analgesics (oral and intravenous) used in the 48 hours was determined by adding intravenous morphine doses to oxycodone/hydrocodone (converted to morphine mg equivalents for analysis with oral oxycodone/hydrocodone 20 mg being considered equivalent to 10 mg intravenous morphine [14]).

^c^NVPS at rest and at movement (defined as sitting upright at 90 degrees) was measured at 6, 24, and 48 hours, and the pain burden was determined as NVPS over time AUC over the 48-hour study period.

^1^Expected postoperative pain: “how much pain do you expect to experience after your surgery on a pain scale of 0–10?”

Anticipated analgesic threshold: “at what point on a pain scale of 0–10 would you likely request postoperative pain relief?”

^2^Perceived analgesic need: “what do you expect your analgesic requirements will be after surgery? (0 = no analgesia, 10 = highest possible amount).”

**(b):** 

Response outcome^a^	*R* ^2^ coefficient	*p* value	Predictor tests in model
Opioid used^b^	0.212	0.0027	2.514 + 0.041 *∗* expected pain^1^
Combined (Rest + Move) NVPS AUC^c^	0.447	0.00001	162.054 + 32.45 *∗* expected pain^1^ – 1.695 *∗* perceived analgesic need^2^

AUC = area-under-the curve; NVPS = numerical verbal pain score (0–10, 0 = no pain, 10 = worst pain imaginable).

^a^Transformations used are as follows.

For MSEqui: anticipated postoperative pain: *x* ^2.4^.

For combined (Rest + Move) NVPS AUC: expected postoperative pain: *x* ^0.9^, perceived analgesic need: *x* ^2.2^.

A total of 38 subjects analyzed (3 and 0 outliers were removed for opioid used and combined NVPS AUC, resp.).

^b^Total amount of supplemental opioid analgesics (oral and intravenous) used in the 48 hours was determined by adding intravenous morphine doses to oxycodone/hydrocodone (converted to morphine mg equivalents for analysis with oral oxycodone/hydrocodone 20 mg being considered equivalent to 10 mg intravenous morphine [14]).

^c^NVPS at rest and at movement (defined as sitting upright at 90 degrees) was measured at 6, 24, and 48 hours, and the pain burden was determined as NVPS over time AUC over the 48-hour study period.

^1^Expected postoperative pain: “how much pain do you expect to experience after your surgery on a pain scale of 0–10?”

Anticipated analgesic threshold: “at what point on a pain scale of 0–10 would you likely request postoperative pain relief?”

^2^Perceived analgesic need: “what do you expect your analgesic requirements will be after surgery? (0 = no analgesia, 10 = highest possible amount).”

**Table 4:** Bivariate correlation coefficients among the preoperative predictive questionnaires.

	ASI	FPQ	PCS	Extroversion	Neuroticism	Lying	Psychoticism	Anticipated pain^1^	Analgesic threshold^2^	Analgesic needs^3^
ASI	1	0.42^†^	0.65^†^	−0.12	0.18	0.21	−0.24	0.12	−0.11	0.2
FPQ	0.42^†^	1	0.57^†^	−0.03	0.08	0.16	−0.56^†^	0.34^*∗*^	0.01	0.15
PCS	0.65^†^	0.57^†^	1	−0.18	0.41^†^	0.03	−0.45^†^	0.29^*∗*^	−0.14	0.14
Extroversion	−0.12	−0.03	−0.18	1	0.07	−0.13	0.06	0.11	0.19	−0.01
Neuroticism	0.18	0.08	0.41^†^	0.07	1	−0.04	−0.08	0.16	0.02	−0.07
Lying	0.21	0.16	0.03	−0.13	−0.04	1	0.13	−0.14	0.07	0.18
Psychoticism	−0.24	−0.56^†^	−0.45^†^	0.06	−0.08	0.13	1	−0.09	0.13	0.06
Expected pain^1^	0.12	0.34^*∗*^	0.29^*∗*^	0.11	0.16	−0.14	−0.09	1	0.25	0.19
Analgesic threshold^2^	−0.11	0.01	−0.14	0.19	0.02	0.07	0.13	0.25	1	−0.01
Analgesic need^3^	0.2	0.15	0.14	−0.01	−0.07	0.18	0.06	0.19	−0.01	1

^†^Adjusted *p* < 0.05, ^*∗*^raw *p* < 0.05.

ASI = Anxiety Sensitivity Index; FPQ = Fear of Pain Score III; PCS = Pain Catastrophizing Scale; Personality categories (psychoticism, extroversion, neuroticism, and lying) were derived from Eysenck Personality Questionnaire Revised-Short Scale (EPQR-S).

^1^Expected postoperative pain: “how much pain do you expect to experience after your surgery on a pain scale of 0–10?”

^2^Anticipated analgesic threshold: “at what point on a pain scale of 0–10 would you likely request post-operative pain relief?”

^3^Perceived analgesic need: “what do you expect your analgesic requirements will be after surgery? (0 = no analgesia, 10 = highest possible amount).”
